# Tillage intensity and pasture in rotation effectively shape soil microbial communities at a landscape scale

**DOI:** 10.1002/mbo3.676

**Published:** 2018-06-13

**Authors:** Cédric Le Guillou, Nicolas Chemidlin Prévost‐Bouré, Battle Karimi, Nouraya Akkal‐Corfini, Samuel Dequiedt, Virginie Nowak, Sébastien Terrat, Safya Menasseri‐Aubry, Valérie Viaud, Pierre‐Alain Maron, Lionel Ranjard

**Affiliations:** ^1^ INRA UMR1347 Agroécologie Dijon France; ^2^ AgroSup Dijon UMR1347 Agroécologie Dijon France; ^3^ INRA UMR1069 Sol Agro et hydrosystème Spatialisation Rennes France; ^4^ INRA UMR1347 Agroécologie Plateforme Genosol Dijon France; ^5^ Université de Bourgogne UMR1347 Agroécologie Dijon France; ^6^ Agrocampus Ouest UMR1069 Sol Agro et hydrosystème Spatialisation Rennes France; ^7^ Université Européenne de Bretagne Bretagne France; ^8^ Present address: Agrosolutions 83 avenue de la Grande Armée 75782 Paris Cedex 16 France

**Keywords:** agricultural practices, bacteria, farmers, fungi, sustainable landuse

## Abstract

Soil microorganisms are essential to agroecosystem functioning and services. Yet, we still lack information on which farming practices can effectively shape the soil microbial communities. The aim of this study was to identify the farming practices, which are most effective at positively or negatively modifying bacterial and fungal diversity while considering the soil environmental variation at a landscape scale. A long‐term research study catchment (12 km^2^) representative of intensive mixed farming (livestock and crop) in Western Europe was investigated using a regular grid for soil sampling (n = 186). Farming systems on this landscape scale were described in terms of crop rotation, use of fertilizer, soil tillage, pesticides treatments, and liming. Molecular microbial biomass was estimated by soil DNA recovery. Bacterial and fungal communities were analyzed by 16S and 18S rRNA gene pyrosequencing. Microbial biomass was significantly stimulated by the presence of pasture during the crop rotation since temporary and permanent pastures, as compared to annual crops, increased the soil microbial biomass by +23% and +93% respectively. While soil properties (mainly pH) explained much of the variation in bacterial diversity, soil tillage seemed to be the most influential among the farming practices. A 2.4% increase in bacterial richness was observed along our gradient of soil tillage intensity. In contrast, farming practices were the predominant drivers of fungal diversity, which was mainly determined by the presence of pastures during the crop rotation. Compared to annual crops, temporary and permanent pastures increased soil fungal richness by +10% and +14.5%, respectively. Altogether, our landscape‐scale investigation allows the identification of farming practices that can effectively shape the soil microbial abundance and diversity, with the goal to improve agricultural soil management and soil ecological integrity.

## 1 INTRODUCTION

Agriculture intensification was developed in the past century to meet the global food demand. It was generally based on highly specialized and intensified annual crop systems based on soil tillage, irrigation, chemical inputs of pesticides and fertilizers, and monospecific crops (Matson, Parton, Power, & Swift, 1997). Although this agricultural system has been largely successful at increasing croplands productivity for over 50 years, it has now been acknowledged to have resulted in a parallel degradation of ecosystems (Millenium Ecosystem Assessment 2005). Most conventional agricultural practices have led to the degradation of natural resources through soil erosion, contamination, decline in above‐ and belowground biological diversity, deforestation, desertification, salinization, and greenhouse gas emissions (Millennium Ecosystem Assessment, 2005). In this context, it is now crucial to develop a more sustainable agriculture that must balance the needs of crop productivity and environment preservation.

A discipline known as Agroecology has emerged in response to new expectations concerning agricultural systems (Altieri, 1989). It introduces the paradigm that for agriculture to be sustainable, the services delivered by biodiversity should be integrated into the management of crop production systems. To pursue such aims, we need to identify and develop farming practices that stimulate the biodiversity related to the diverse agroecosystem services. For a few decades, certain agricultural practices such as crops diversification, use of cover‐ and intercrops, tillage reduction, and organic fertilization have been experimentally demonstrated to improve soil fertility (e.g., Lienhard et al., 2013; Steiner et al., 2007). Nevertheless, they now need to be evaluated at the complex scale of farming systems.

Agricultural production relying on soil biologically derived functions may provide a model system for ecological intensification of agriculture (Giller, Beare, Lavelle, Izac, & Swift, 1997). Among the different options, management of microbial diversity seems promising since soil microorganisms play a vital role in agroecosystems due to their participation in a wide range of services (Kennedy & Smith, 1995). Soil microbial diversity is considered to be critical to the integrity, function, and long‐term sustainability of agroecosystems (Maron, Mougel, & Ranjard, 2011). Promoting the taxonomic and functional diversity of microorganisms may have beneficial effects for soil nutrients cycling (Philippot et al., 2013; Tardy et al., 2015), pathogen management (Vivant, Garmyn, Maron, Nowak, & Piveteau, 2013) and soil functions stability to environmental changes (Tardy et al., 2014). Therefore, determining the agricultural management practices able to modify microbial diversity is important for selecting sustainable farming practices.

Many studies found that soil physicochemical properties (e.g., pH, texture) were the dominating drivers of microbial communities (e.g., Bru et al., 2011; Dequiedt et al., 2011; Fierer & Jackson, 2006). However those studies were conducted at the continent, country or regional scale. It is probable that the weak response to management practices can be explained by the large variation in pedoclimatic characteristics. Altogether those studies highlight the need to investigate the landscape scale to better identify the farming practices able to shape microbial communities. Besides, the landscape is a relevant scale for cropland management by farmers and for the implementation of agricultural strategies by decision‐makers. Some studies dealt with the agricultural landscape scale but evaluated basic land management categories such as forests versus grasslands versus croplands (e.g., Constancias, Saby, et al., 2015; Constancias, Terrat, et al., 2015). We now need to quantitatively integrate the range of farm systems variation and identify those farming practices which have the most impact. Moreover, studies at landscape scale often focused on the bacterial community (e.g., Constancias, Saby, et al., 2015; Constancias, Terrat, et al., 2015; Landa et al., 2014). However, the fungal community also has a major role in agroecosystem functioning and needs to be addressed (De Boer, Folman, Summerbell, & Boddy, 2005).

In this context, the aim of our study was to identify and rank farming practices shaping the indigenous soil bacterial and fungal communities. A 12‐km^2^ agricultural landscape covering a range of intensive farming practices (combined livestock and crop/pasture systems) was investigated and quantitatively and qualitatively described. Such an agricultural landscape approach was used as (1) it provides a range of actual farming practices and soil characteristics that may reveal how microbial communities can be effectively shaped and (2) it represents a relevant and realistic scale of agricultural management. Soil physicochemical properties and molecular microbial biomass were characterized as well as bacterial and fungal diversity through high‐throughput sequencing analysis. We mapped all these microbial parameters at this landscape scale and hypothesized that agricultural practices would be better identified and ranked according to their effects on soil microbiota than in previous studies at broader scales due to the smaller variation in soil characteristics.

## 2 MATERIAL AND METHODS

### 2.1 Landscape description

**Figure 1:**
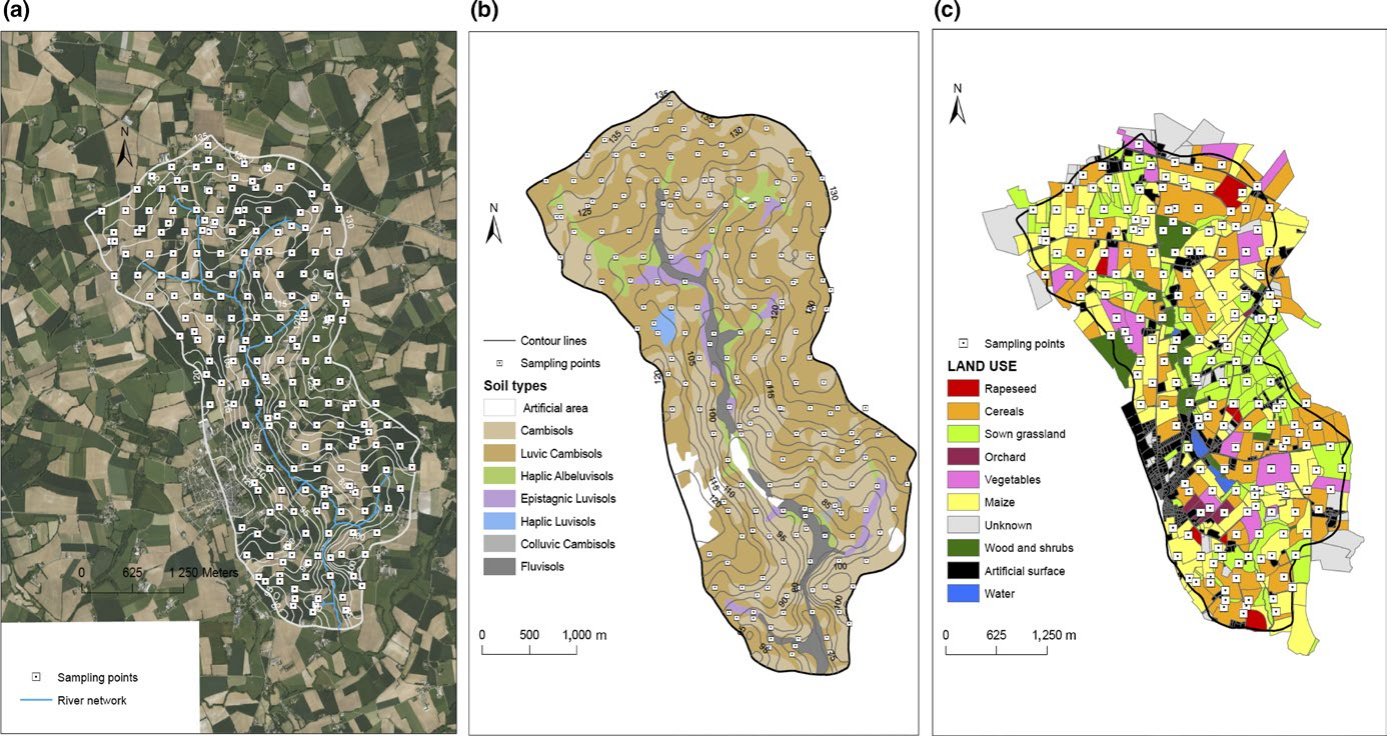
Maps of the Naizin landscape indicating (a) the locations of the sampling points, (b) the variations of soil types, (c) the variations of land uses

The study was carried out on a 12‐km^2^ agricultural landscape located in Brittany, Western France (Latitude: 48°00′ N, Longitude: 2°49′ O), 85% of which is dedicated to agriculture (Figure 1). This is a long‐term research study site part of a French network of agricultural catchment research (SOERE RBV, focused on the Critical Zone) (http://www6.inra.fr/ore_agrhys). The catchment is representative of the intensive mixed agricultural landscape of Western Europe with a temperate climate. The site is heterogeneous in terms of cropping systems, environmental conditions, and has a long agricultural past (Figure 1). The farming systems involve intensive livestock (pig, dairy cattle, poultry) and crop production (annual crops (cereals, vegetables), pasture). Land‐use history and farming practices have been documented since 1993 (Payraudeau, Van Der Werf, & Vertes, 2007) and were updated for the present study through a farmers survey. The soils are silty loam and range from Cambisol to Luvisol and Fluvisol (Walter & Curmi, 1998; WRB 2006) (Figure 1). Parent material consists of schist and fluvial deposits in bottom land. The soil water regime was defined by drainage class based on the soil's description. According to the study by Walter and Curmi (1998), soils vary mainly in their water regime characteristics and are organized according to topography. The soil system comprises a well‐drained upland domain and a poorly drained bottomland where the soils are temporarily or permanently saturated.

Numerous studies on hydrogeochemical transfers, C and N cycles, and multicriteria evaluation of the agroecosystems, have been conducted on this site (Durand et al., 2010; Payraudeau et al., 2007).

### 2.2 Soil sampling

The sampling design covered the entire agricultural landscape and was based upon a regular triangular grid with spacing of 250 m, which corresponds to 199 georeferenced sites (WGS 84 GPS coordinates) (Figure 1). Some sites corresponded to uncultivated land (e.g., forest, wetland), that brings the number of agricultural sites actually examined in the present study to 186. All sites were sampled during the last week of June 2013. The sample period was defined to ensure that no farming practices had recently occurred (e.g., organic fertilization). At each site, five soil subsamples (and a soil core for bulk density analysis) were taken at 0–15 cm depth over a surface of 1 m^2^ in the interrow. Subsamples were bulked, sieved at 4 mm, and divided for the different analyses. Samples were kept at 4°C throughout the field soil sampling campaign. Soil samples for microbial analysis were sieved at 2 mm, lyophilized at −80°C, and stored at −40°C. Soil samples for physicochemical analysis were air‐dried and stored at room temperature.

### 2.3 Farming practices characterization

The cropping systems were accurately characterized over the landscape by surveying farmers and integrating the past surveys conducted since 1993. The main farming practices were evaluated and described according to diverse criteria (Table 1). Cropping systems were described in terms of crop rotation (length, crop type, yields, and annual crop residue management), fertilization (organic and mineral amounts, organic type, and frequency), tillage (depth and frequency), liming (amount and frequency), and pesticides treatments (type and frequency). In this study, eight variables representing these main farming practices were selected for subsequent variance partitioning statistical analysis (Table 1). This selection was based on the exclusion of collinear variables, maximum representativeness, and homogeneous values distribution across the 186 samples (as some practices were carried out by only a few farmers).

**Table 1:** Description of the farm management practices determined through the survey

Farm management	Description	Selected variables	Unit	[min; max]	CV
Crop rotation	Rotation length and crop type	Crops diversity	Number	[1; 8]	39.7
		Residues input	Ton of C/year/hectare	[0; 6.31]	37.3
		Pasture frequency	Number/year	[0; 1]	113.1
		Legume presence	Yes; no	na	na
Fertilization	Type. quantity and frequency	Organic fertilization	Ton of C/year/hectare	[0; 2.84]	76.2
Tillage	Depth and frequency	Ploughing	Number/year	[0; 1.61]	51.4
CaO input	Quantity and frequency	Liming	kg of CaO/year/hectare	[0; 699.58]	111.7
Pesticide	Number of herbicide. fungicide and insecticide treatments	Pesticides	Number/year	[0; 4.47]	61.3

*Note*. The eight selected variables for subsequent variance partitioning statistical analysis were chosen for representing the main farming practices and based on the exclusion of collinear variables. Maximum representativeness and homogeneous values distribution across the 186 samples. (na = nonapplicable. CV = coefficient of variation).

### 2.4 Soil analyses

#### 2.4.1 Physicochemical properties

The main soil physicochemical properties were determined by the INRA Soil Analysis Laboratory (France, Arras) according to standard international procedures. Soil texture was determined according to the NF X 31‐107 standard. The pH in water was determined after soil suspension in water (1:5 ratio). Organic carbon (C org) and total nitrogen (N tot) were determined by dry combustion. Phosphorus was determined for its total (HF–HClO4 extraction) and available (Dyer method, NF X 31‐60) fractions. Copper was determined following EDTA extraction. Aluminum, silica, and iron were determined following the Tamm extraction. Soil bulk density was evaluated using oven‐dried (24 hr, 105°C) soil cores sieved at 2 mm to discard the stone fraction.

#### 2.4.2 Microbial communities characterization

##### Soil DNA extraction and molecular microbial biomass quantification

Soil DNA was extracted and purified at the INRA Genosol Platform (France, Dijon) using the procedure described by Plassart et al. (2012). Crude DNA extracts were quantified by gel agarose electrophoresis, stained with ethidium bromide using calf thymus DNA as a standard curve, and used as a reliable estimate of soil microbial biomass (Dequiedt et al., 2011). After quantification, crude DNA samples were purified on PVPP (polyvinylpolypyrrolidone) Microbiospin minicolumns (Bio‐Rad). The eluates were then collected and purified for residual impurities using the Geneclean Turbo Kit (MP‐Biomedicals, NY, USA). The purified DNA extracts were quantified using the Quantifluor staining kit (Promega, Madison, Wisconsin, USA).

##### Pyrosequencing of 16S and 18S rRNA gene sequences

Bacterial diversity was estimated by 454 pyrosequencing of the 16S rRNAV3–V4 gene region as described by Terrat et al. (2012). A gene fragment (about 400 bp) was first amplified using the primers F479 and R888. A second PCR was run on purified PCR products using 10 based‐pair multiplex identifiers (MID) added at 5′ position to the primers for subsequent sample identification. The PCR products were finally purified and pyrosequenced on a GS Roche 454 Sequencing System. Fungal diversity was similarly estimated by 454 pyrosequencing of the 18S rRNA. A gene fragment (about 350 bp) was first amplified using the primers FR1 and FF390. The subsequent steps to pyrosequencing were similar to the bacterial analysis described earlier.

##### Bioinformatics analyses

Bioinformatics analysis of the generated sequences was performed using the GnS‐Pipe of the INRA Genosol Platform as described by Terrat et al. (2012). 16S and 18S raw reads were sorted according to the multiplex identifier sequences. Raw reads were then filtered based on their length, number of ambiguities, and primers sequences. To avoid biased community comparison, the retained reads were homogenized by random selection close to the lowest dataset (2,886 and 3,286 high‐quality sequences for 16S and 18S rRNA genes, respectively). A PERL program was applied for rigorous dereplication (i.e., clustering of strictly identical sequences). Dereplicated reads were aligned using infernal alignment and clustered at 95% similarity into operational taxonomic units (OTU) using a PERL program that groups rare reads to abundant ones and does not count differences in homopolymer lengths. A filtering step was then carried out to check all single singletons (reads detected only once and not clustered, which might be artifacts, such as PCR chimeras) based on the quality of their taxonomic assignments. Finally, contingency tables of OTUs were obtained for both bacteria (16S) and fungi (18S) with the samples in lines and OTUs in columns, indicating the number of reads in each OTU for all samples. For each sample, the number of bacterial and fungal OTUs corresponded to bacterial and fungal richness; respectively. Bacterial and fungal evenness corresponded to Pielou's index.

Bacterial and fungal diversities were characterized by OTU richness and evenness.

### 2.5 Mapping using geostatistics

A geostatistical method was used to map the microbial biomass, bacterial richness and fungal richness and to characterize their spatial variations (https://doi.org/10.5281/zenodo.1063500). When the data did not follow a normal distribution, log or squared transformation was applied before modeling the spatial correlations. In conventional geostatistical analysis, an estimate of a variogram model is computed based on the observations, which describe the spatial variation of the property of interest. This model is then used to predict the property at unsampled locations using kriging (Webster & Oliver, 2007). A common method for variogram estimation is first to calculate the empirical (so called experimental) variogram by the method of moments (Matheron, 1965), and then to fit a model to the empirical variogram by (weighted) nonlinear least squares. We tried to fit several models and retained the one that minimized the objective function (Minasny & McBratney, 2005). The validity of the best fitted geostatistical model was then assessed in terms of the standardized squared prediction errors (SSPE) using the results of a leave one out cross validation. If the fitted model provided a valid representation of the spatial variation of the microbial biomass or richness, then these errors would have a χ² distribution with a mean of 1 and median of 0.455 (Lark, 2002). The mean and median values of the SSPE were also calculated for 1,000 simulations of the fitted model to determine the 95% confidence limits and to obtain a map of kriging standard error (data not shown). The geostatistical analysis gstat package was used for variograms analysis and kriging. The effective range of the variograms fitted on the data represents the size of the geographical patches.

### 2.6 Statistical analyses

Two statistical approaches were carried out. One based on the analysis of the overall effects of farming practices using categorical classification of farming practices (Table 3). The other one was based on the variance partitioning analysis (Table 4), using the variables selected in Table 1, to rank the farming practices according to their influence on microbial communities.

Overall effects of farming practices on the different characteristics of the microbial communities were evaluated by Kruskal–Wallis (for crop types) and Mann–Whitney nonparametric tests. Pasture was defined as being permanent when the pasture was cropped for at least six consecutive years, and temporary when the pasture was cropped between 1 and 5 years (Huyghe et al., 2005). Low and high groups of the other farming practices were determined by splitting the dataset in two at the median value of the specific practice considered. Calculations were performed with XLSTAT (Addinsoft, Paris, France).

The relative contributions of soil physicochemical properties and farming practices in shaping the microbial communities characteristics were evaluated by variance partitioning. In total, eight farming practices (Table 1) and eight soil physicochemical properties were selected as explanatory variables of microbial biomass, and bacterial and fungal diversity indexes (explained variables). Selection of these explanatory variables was based on the exclusion of collinear variables (*r* < 0.7), maximum representativeness, and homogeneous values distribution across the 186 samples (as some variables were represented in only a few samples). These selection steps are necessary criteria for accurate model prediction (Ramette, 2007). The selected physicochemical properties were: bulk density, drainage class, *x* coordinate (used as a synthetic descriptor of samples position in the landscape), pH, clay, C org, *C*/*N*, and available *P*. Data were standardized to approximate a Gaussian and homoscedastic residual distribution. The variables significantly shaping microbial communities were determined by applying a stepwise selection procedure to all physicochemical and farming practices variables by maximizing the adjusted *r* ^2^ while minimizing the Akaike Information Criteron (Ramette, 2007). The respective amounts of variance (i.e., marginal and shared) were determined by canonical variation partitioning and the adjusted *r* ^2^ with RDA (Ramette, 2007). The statistical significance of the marginal effects was assessed from 999 permutations of the reduced model. All these analyses were performed with R‐free software (http://www.r-project.org/) using the vegan package (Oksanen et al., 2011).

## 3 RESULTS

### 3.1 Landscape variation in farming practices and soil physicochemical properties

At the sampling time, crop types ranged from permanent pasture (n = 24) to temporary pasture (n = 74) and annual crops (n = 88). Farming practices differed in terms of organic fertilization (0–2.84 Mg C/year) and annual crop residues incorporation (0–6.31 Mg C/year). They also differed in terms of pesticides and tillage intensity, with 0–4.47 and 0–1.61 interventions/year, respectively. Use of liming varied greatly and ranged from 0 to 699.58 kg CaO input/year. A legume was sometimes introduced into the rotation (n = 37). Coefficients of variation of the selected variables (Table 1) were ordered as follows: pasture frequency > CaO input > organic fertilization > pesticides treatments frequency > tillage frequency > crops diversity > annual crop residues input.

Summary statistics for the physicochemical characteristics of the 186 soils sampled across the studied agricultural landscape are shown in Table 2. Soils were homogeneous in terms of texture with a predominant silty fraction (mean = 65.1%). According to the USDA classification, most soils were categorized as silt loam (two soils were categorized as silty clay loam). Soils were slightly acid (mean pH = 6.0) and exhibited very low variations (CV = 7.5%). Organic carbon and total nitrogen were highly correlated (*r* ^2^ = 0.98, *p* < 0.001) and ranged from 14.6 to 72.3 g/kg soil and 1.4 to 6.2 g/kg soil, respectively.

**Table 2:** Summary statistics of physicochemical and microbial soil characteristics across the investigated agricultural landscape (n = 186)

	Mean (SD)	Median	[min; max]	CV
Soil bulk density (g·m^3^)	1.1 (0.2)	1.1	[0.5; 1.5]	14.0
Clay (%)	17.6 (2.5)	17.2	[13.5; 35.6]	14.3
Silt (%)	65.1 (4.8)	65.9	[50.9; 74.4]	7.3
Sand (%)	17.5 (4.2)	16.7	[7.7; 30.4]	24.2
Organic carbon (g/kg)	27.6 (8)	26.3	[14.6; 72.3]	28.9
Total nitrogen (g/kg)	2.4 (0.7)	2.3	[1.4; 6.2]	28.1
*C*/*N*	11.2 (0.7)	11.2	[9.7; 12.7]	6.1
pH water	6.0 (0.4)	6.0	[4.6; 7.4]	7.5
Available *P* (g/kg)	0.6 (0.3)	0.6	[0.02; 1.9]	50.8
Total *P* (%)	0.3 (0.07)	0.3	[0.1; 0.6]	25.2
Cu EDTA (mg/kg)	4.9 (2.7)	4.4	[1.1; 14.3]	54.2
Fe (%)	0.5 (0.1)	0.5	[0.1; 1.3]	24.9
Al (%)	0.3 (0.08)	0.3	[0.1; 0.6]	27.1
Si (%)	0.05 (0.01)	0.04	[0.01; 0.1]	25.4
Microbial biomass (μg DNA/g)	59.2 (34.5)	52.6	[11.8; 251.7]	58.3
Bacterial richness	760 (56)	763	[573; 884]	7.0
Bacterial evenness	0.87 (0.01)	0.87	[0.802; 0.894]	1.3
Fungal richness	476 (93)	461	[235; 742]	20.0
Fungal evenness	0.66 (0.04)	0.67	[0.551; 0.749]	5.8

### 3.2 Landscape distribution of soil microbial characteristics

The amount of soil microbial biomass ranged from 11.8 to 251.7 μg DNA/g soil and showed the highest coefficient of variation (CV = 58.3%) among the different properties analyzed across the landscape (Table 2). Diversity indexes calculated from the pyrosequencing data showed that bacterial richness and evenness ranged from 573 to 884 OTU and from 0.802 to 0.894, respectively. Fungal richness and evenness ranged from 235 to 742 OTU and from 0.551 to 0.749, respectively. The rarefaction curves of bacterial and fungal OTU confirmed that our sequencing effort allowed an accurate description of the bacterial diversity in each soil sample (data not shown).

**Figure 2:**
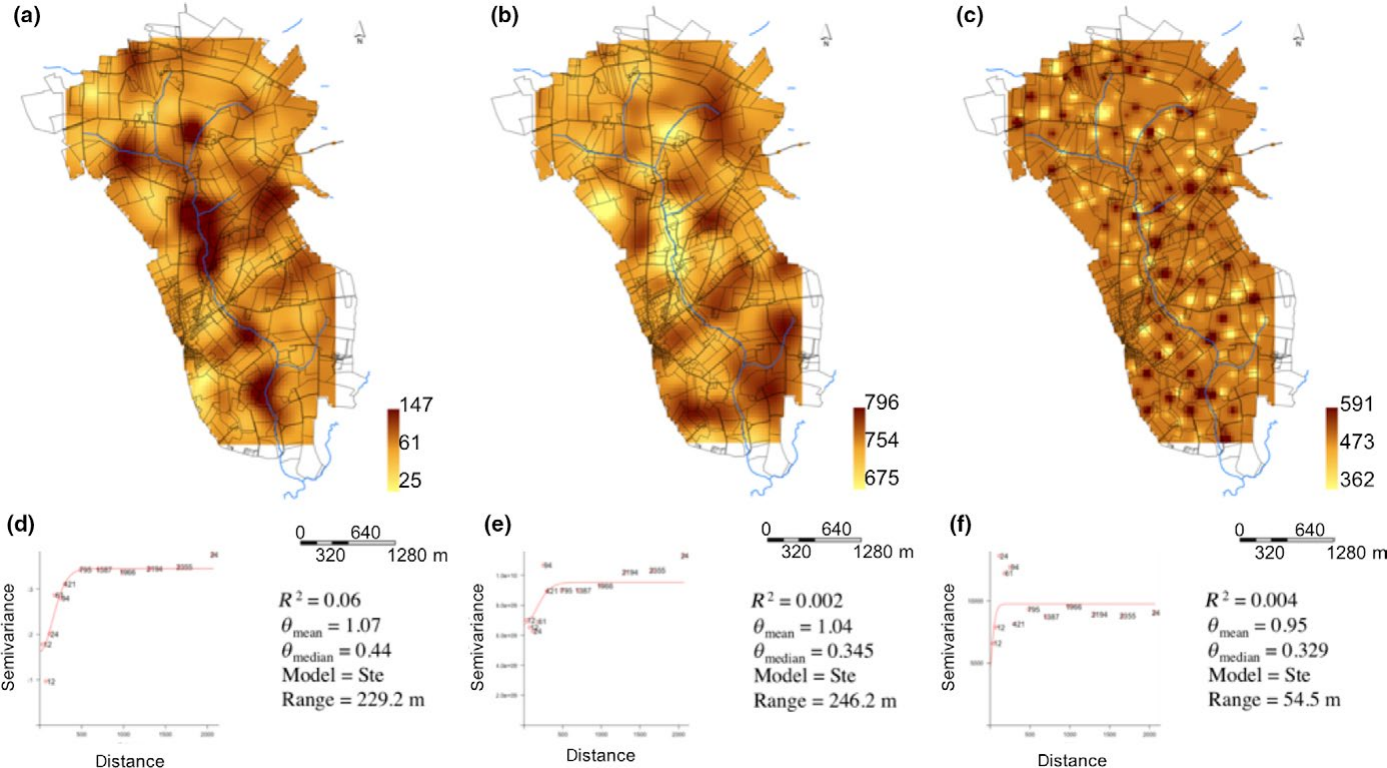
Mapping of microbial descriptors across the studied agricultural landscape. (a–c) Maps of microbial molecular biomass (μg/g soil), bacterial richness (number of OTUs) and fungal richness (number of OTUs). (d–f) Variograms and parameters of kriging models for the three microbial descriptors

The geostatistical approach lead to a robust representation of the spatial variations of soil molecular microbial biomass, bacterial and fungal richness through. Indeed, the mean and median of the distribution of standardized squared prediction errors (θ_mean_, θ_median_) were close to targeted values (1 and 0.455, Lark, 2002). Visual examination of maps evidenced systematically a heterogeneous distribution of soil molecular microbial biomass, bacterial and fungal richness but with different distribution patterns (Figure 2a–c). Molecular microbial biomass and bacterial richness were distributed in less numerous and larger patches than fungal richness. More precisely, fitted models gave an effective range of 229, 246, and 54.5 m for microbial biomass, bacterial and fungal richness, respectively. In addition, the results of the cross validation confirmed the validity of the spatial predictions for microbial biomass but to a lesser extent for bacterial and fungal richness (Figure 2d–f). Moreover, the nugget effect of soil molecular microbial biomass (47.1%), bacterial richness (70.8%) and fungal richness (48.2%) suggest that a part of the spatial variability of soil microbial characteristics may appear at spatial scales smaller than the 200 m.

### 3.3 Overall effects of farming practices on soil microbial characteristics

Temporary and permanent pastures had a significantly higher microbial biomass, a significantly lower bacterial evenness (*p* < 0.01), and a higher fungal evenness or fungal richness (*p* < 0.001) than annual crops (Table 3). Bacterial richness did not differ significantly between temporary and permanent pastures and annual crops. High annual crop residues input and organic fertilization had no effects on microbial characteristics. High tillage had significant negative effects on microbial biomass and fungal evenness (*p* < 0.001) and tended to decrease fungal richness. Inversely, it had significant positive effects on bacterial evenness (*p* < 0.01) and bacterial richness (*p* < 0.05). High pesticides treatments had significant negative effects on microbial biomass and fungal evenness (*p* < 0.001) and tended to decrease fungal richness. The presence of a legume in the rotation had a weak positive effect on the microbial biomass. High liming input had a significant positive effect on bacterial richness (*p* < 0.05) and a negative effect on fungal evenness (*p* < 0.05).

**Table 3:** Mean values of the effects of the different farming practices on the soil microbial biomass and bacterial and fungal diversity indexes

	Crop types				Organic fertilization			Crop residues input			Tillage			Pesticides			Legume presence in rotation			Liming		
	Annual crops	Temporary pasture	Permanent pasture	*p*	Low	High	*p*	Low	High	*p*	Low	High	*p*	Low	High	*p*	Yes	No	*p*	Low	High	*p*
Microbial biomass	48.8 a	60.2 b	94.2 b	**	63.1	54.7	ns	63.0	56.1	ns	67.8 b	49.1 a	***	69.1 b	49.1 a	***	64.6	57.8	ns	58.7	59.6	ns
Bacterial richness	762	765	736	ns	762	758	ns	762	757	ns	752 a	770 b	*	755	765	ns	758	761	ns	751 a	768 b	*
Bacterial evenness	0.868 b	0.867 ab	0.856 a	**	0.866	0.866	ns	0.866	0.866	ns	0.863 a	0.869 b	**	0.864	0.868	ns	0.865	0.866	ns	0.864	0.867	ns
Fungal richness	449 a	494 b	514 b	**	477	474	ns	472	478	ns	486	464	ns	486	466	ns	476	476	ns	488	464	ns
Fungal evenness	0.649 a	0.674 b	0.685 b	***	0.663	0.664	ns	0.661	0.665	ns	0.675 b	0.651 a	***	0.672 b	0.655 a	***	0.667	0.663	ns	0.671 b	0.657 a	*

*Note*. Values with different letters differ significantly. Significance level is indicated as follows: *p < 0.05. **p < 0.01. ***p < 0.001. ns, nonsignificant.

### 3.4 Ranking drivers of soil microbial communities characteristics

**Figure 3:**
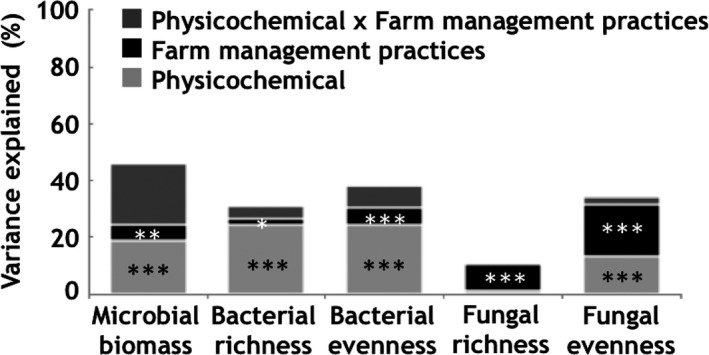
Variance partitioning of the molecular microbial biomass, and bacterial and fungal diversity variables as a function of soil physicochemical and farming practice factors (and their interactions). The amount of explained variance corresponds to the adjusted *r* ^2^ values of the contextual groups using partial redundancy analysis. The significance level of the contribution of the sets of variables is indicated as follows: *p < 0.05, **p < 0.01, ***p < 0.001, ns = nonsignificant

Variance partitioning was used to evaluate and rank the relative contributions of the main soil properties and farming practices influencing the microbial communities’ characteristics. The total amount of explained variance ranged from 10.8% (fungal richness) to 45.7% (microbial biomass) (Figure 3). The analysis showed that farming practices had a systematic significant effect on the variation of microbial characteristics and were the main drivers of soil fungal richness (9.5%, *p* < 0.001) and evenness (18.6%, *p* < 0.001). Inversely, soil physicochemical properties were the main drivers of bacterial richness (24.4%, *p* < 0.001) and evenness (24.6%, *p* < 0.001). Interactions between soil physicochemical properties and farming practices explained relatively small amounts of variance except for soil microbial biomass. The main driver of microbial biomass was the interaction between soil physicochemical properties and farming practices (21.5%).

The relative contribution of each variable, within the sets of soil physicochemical properties and farming practices, was ranked according to the respective amounts of variance explained (Table 4). Their positive or negative effect on microbial biomass and diversity indexes was also indicated (positive or negative sign in Table 4). SOC (15.4%, *p* < 0.001) and *C*/*N* (4.1%, *p* < 0.001) were the main predictors of microbial biomass (in a positive and negative sense, respectively). Frequency of pasture during the crop rotation was the only significant farming practice variable selected in the model (1.9%, *p* < 0.05) and was positively related to the microbial biomass. Soil pH was the main predictor (positive sense) of bacterial richness (17.1%, *p* < 0.001) and evenness (14%, *p* < 0.001). Soil tillage was the only farming practice variable (positive sense) significantly selected in the model to explain the bacterial richness (2.3%, *p* < 0.05) and evenness (5.6%, *p* < 0.001). Among the explanatory variables, pasture frequency was the only predictor (positive sense) of fungal richness (9.5%, *p* < 0.001) and the main predictor of fungal evenness (18.5%, *p* < 0.001). The variation in fungal evenness was also noticeably explained by a number of physicochemical properties: soil bulk density (4.5%, *p* < 0.01), drainage class (2.6%, *p* < 0.05), *x* coordinate (2.1%, *p* < 0.01), and soil available *P* (2.0%, *p* < 0.05).

**Table 4:** Variance explained (%) of the determined microbial biomass and bacterial and fungal diversity indexes across the agricultural landscape

	Microbial biomass		Bacterial richness		Bacterial evenness		Fungal richness		Fungal evenness	
	Explained variance (%)	Relation	Explained variance (%)	Relation	Explained variance (%)	Relation	Explained variance (%)	Relation	Explained variance (%)	Relation
Physicochemical										
*C*/*N*	4.1%***	–								
Clay					2.5%*	–				
Hydromorphia			1.7%*	+					2.6%*	+
pH water	1.5%*	+	17.1%***	+	14%***	+				
SOC	15.4%***	+								
Soil available *P*									2.0%*	–
Soil bulk density			3%*	–	3.1%**	–			4.5%**	+
*x* coordinate			3.2%**	+					2.1%**	–
Farm management practices										
Crop residues input										
Legume presence in the rotation									1.9%*	–
Pasture frequency	1.9%*	+					9.5%***	+	18.5%***	+
Organic fertilization										
Pesticides										
Soil tillage			2.3%*	+	5.6%***	+				

*Note*. Missing values indicate that the variable was not retained in the model. Significance level is indicated as follows: *p < 0.05. **p < 0.01. ***p < 0.001. ns = nonsignificant.

## 4 DISCUSSION

Most studies dealing with the effects of agricultural management on soil microbial communities have been conducted at field scales in station‐based experiments with very few comparisons of practices. However, the genericity of such an approach is weak, considering the variations in soil physicochemical characteristics and agrosystems management strategies currently existing at the landscape scale. As a consequence, information about the ability of agrosystems management options to effectively shape microbial communities is lacking. Here, we determined the influence of farming practices on the diversity of indigenous soil bacteria and fungi at a landscape scale.

### 4.1 Landscape soil properties and farming practices

Soils from the studied landscape were homogeneous and representative of the Brittany region. As expected, they had a silty texture and a slightly acid pH (mean = 6.0) with little variation at this landscape scale. Their contents of organic carbon (mean = 27.6 g/kg), total nitrogen (mean = 2.4 g/kg), and available *P* (mean = 0.6 g/kg) were high. This is in agreement with Walter and Curmi (1998) who related soil properties to the pedoclimatic conditions and farming practices (e.g., organic fertilization) on this site.

Agricultural management over the investigated landscape differed primarily according to the cropping systems (annual crops, temporary, and permanent pastures). A highly contrasting use of liming was also apparent. Variations in organic fertilization, tillage and pesticides treatments were of secondary importance. Therefore, as expected, the agricultural landscape investigated in the present study showed variability in farming practices and relatively little variation in soil characteristics. This landscape approach enabled us to effectively identify the farming practices most able to shape bacterial and fungal diversity.

### 4.2 Soil characteristics as drivers of microbial communities

Geostatistical predictions of soil microbial biomass and diversity evidenced a heterogeneous distribution but, with different size range for each microbial parameter. As indicated by the variogram parameters, the spatial structuration of microbial biomass is more significant than for bacterial and fungal richness. In addition, visual comparison of the different maps evidenced that the location of the hot and low spots was different for each microbial parameter and suggested that these variations were related to soil characteristics (as represented by soil types above). Altogether, these observations lead to conclude that various environmental factors drive microbial biomass and diversity as previoulsy observed at the landscape scale (Constancias, Saby, et al., 2015; Constancias, Terrat, et al., 2015.

In this way, our study confirmed the importance of soil properties as drivers of microbial communities (Constancias, Saby, et al., 2015; Constancias, Terrat, et al., 2015). Similar results have been obtained at national (Dequiedt et al., 2011; Griffiths et al., 2011) and continental scales (Fierer & Jackson, 2006). However, at our scale of investigation, soil properties drove the microbial biomass and bacterial diversity indexes in particular, as they represented most of the explained variance, but did not drive the fungal diversity indexes. The importance of soil properties in determining microbial variables was as follows: Bacterial richness ≥ Bacterial evenness > Microbial biomass > Fungal evenness > Fungal richness.

More precisely, soil organic carbon content and quality (*C*:*N* ratio) were the main determinants of the microbial biomass. This observation is in accordance with the results of Constancias, Saby, et al., 2015; Constancias, Terrat, et al., 2015 also obtained at a landscape scale. The soil organic matter content and its recalcitrance to degradation were also related to the abundance of microorganisms in field experiments or at a wider scale (De Boer et al., 2005).

pH was the overriding driver of bacterial diversity, explaining most of the variance in the bacterial diversity indexes, despite its low range of variation at our landscape scale. The importance of pH as a bacterial diversity driver has previously been shown from continental and national scales down to the landscape scale and also at the soil aggregate scale (Constancias et al., 2014; Constancias, Saby, et al., 2015; Constancias, Terrat, et al., 2015; Griffiths et al., 2011). In addition, Griffiths et al. (2016) compiled a predictive map of soil bacterial community structure at the European scale based solely on this soil parameter.

Our study at a landscape scale showed that the variation in bacterial characteristics could be partly explained by some other soil properties (bulk density, clay content, drainage class, *x* coordinate). In agreement with studies that revealed soil texture and structure, soil water regime and geoposition to be drivers of microbial communities (Castellanos, Dohrmann, Imfeld, Baumgarte, & Tebbe, 2009; Drenovsky, Steenwerth, Jackson, & Scow, 2010; Hu et al., 2014; Landa et al., 2014), we showed that they also partly operate at an agricultural landscape scale. The soil physicochemical properties selected in the model (soil water regime, available *P*, bulk density, SOC, x coordinate) have ever been found to influence the fungal community (De Vries et al., 2012; Lauber, Strickland, Bradford, & Fierer, 2008; Watts, Torbert, Feng, & Prior, 2010) but were of relatively less importance than farming practices in the present study.

### 4.3 Farming practices as drivers of soil microbial communities

We first used nonparametric tests for overall comparison of the samples (categorical analysis) and to provide a general insight into the potential effects of farming practices (analyzed separately) on microbial characteristics while acknowledging that some soil characterictics and practices may covary and that some practices may have confounding effects. Nevertheless, visual comparison of the different maps also suggested that variations in each microbial parameter were related to land use and associated farming practices. Particularly, the size of the patches was in the range of that of agricultural plots.

Our analysis revealed an increase in microbial biomass from annual crops to permanent pastures with a significantly higher microbial biomass in temporary and permanent pastures than in annual crops. Interestingly, among all the soil microbial properties, microbial biomass showed the highest coefficient of variation, and the value obtained from the 186 soils across the present landscape was equivalent to that of the 2,150 soils in the French national monitoring network (RMQS) (Dequiedt et al., 2011). It supports the considerable variation in microbial biomass at different scales, and thus its potential use as a bioindicator of soil agricultural management (Horrigue et al., 2016). Organic inputs such as annual crop residues and organic fertilization did not have any effects on the soil microbial biomass. The rates of these organic inputs (annual crop residues and organic fertilization) may be too low considering the relatively constrained changes in landscapes with complex interactions between farming practices and soil properties. In addition, the amounts of organic input may be small in relation to the high soil organic C contents. High tillage and pesticides treatment frequency both decreased the microbial biomass and could be potential management levers. Their negative effects have been previously observed (Karlen et al., 1994; Perucci, Dumontet, Bufo, Mazzatura, & Casucci, 2000). Considering the limits of such global analyses, we then used variance partitioning to identify and rank the marginal effects of soil properties and farming practices. Variance partitioning revealed that farming practices and soil physicochemical properties interacted to determine the microbial biomass. However, the variance partitioning analysis confirmed that a significant agricultural option for managing microbial biomass was the presence of pastures during the crop rotation. A pasture increases the soil organic matter content (Chan et al., 2011) and consequently increases the microbial biomass (Horrigue et al., 2016). Maintaining a soil cover enables constant carbon flow into the soil (through exudates, and recycling of roots and plant residues), which may be more efficient at durably increasing the microbial biomass than occasional organic inputs such as annual crop residues or organic fertilization.

The bacterial diversity results showed that richness was positively affected by high tillage intensity and liming and that evenness was also positively affected by high tillage intensity and crop types (it was significantly higher in annual crops than in permanent pastures). The observed liming effects are probably related to combined effects on pH and cations availability (as there was no significant relationship between pH and liming). Variance partitioning analysis confirmed the significant influence of tillage on bacterial richness and evenness. Positive effects of tillage on bacterial diversity have been previously observed (Lienhard et al., 2014). In agreement with Constancias, Saby, et al., 2015; Constancias, Terrat, et al., 2015, the greater effects of tillage on bacterial evenness, rather than on richness, suggest that soil physicochemical characteristics influence the number of species and that agricultural management modulates the population equilibrium. Overall, our results demonstrate that soil tillage is the most important in such farming systems for shaping bacterial diversity.

The fungal diversity results showed that richness and evenness increased from annual crops to permanent pastures (the indexes being significantly higher in permanent and temporary pastures than in annual crops). Evenness was further negatively affected by soil tillage, pesticide treatments, and liming. The effects of these factors have already been reported and the fungal community has often been related to soil nutrients status and disturbance intensity (e.g., Drenovsky et al., 2010; Lauber et al., 2008; Lienhard et al., 2014). Variance partitioning revealed that farming practices, in contrast to their effects on the bacterial community, were the dominant drivers of fungal richness and evenness variation. Variance partitioning analysis indicated that the presence of pastures during the crop rotation was the most important factor influencing the fungal diversity indexes. The greater effect of pasture on fungal evenness rather than richness suggests that agricultural management mainly modulates the population equilibrium. Overall, our study demonstrates that the presence of pastures during the crop rotation is the most able to shape the fungal diversity in farming systems including both cropping and breeding activites.

## 5 CONCLUSIONS

We demonstrated that actual farming practices can effectively shape soil microbial communities in terms of abundance and richness diversity. Some practices have a more significant effect than others and differ in their shaping of microbial biomass, bacterial, or fungal diversity. Tillage and presence of pastures during the rotation were the most influential in shaping the overall soil microbial communities in these complex farming systems. We propose that cropping systems with temporary pastures could represent intermediate situations regarding the promotion of microbial diversity and could model systems with fair trade‐off between the need for agriculture productivity and ecological integrity preservation. The results of this research will ultimately contribute to design landscape management strategies for precision agriculture with rearrangements in the spatial organization for an optimal location of cropping systems in landscape to best suit the constraints and opportunities of the soil resource. Further investigation integrating microbial community composition and the identification of particular dominant species could be relevant to refine the impact of farming practices on soil microbila quality and functioning.

## CONFLICT OF INTEREST

The authors declare no conflict of interest.
